# Virtual training, real effects: a narrative review on sports performance enhancement through interventions in virtual reality

**DOI:** 10.3389/fpsyg.2023.1240790

**Published:** 2023-10-19

**Authors:** Fabio Richlan, Moritz Weiß, Patrick Kastner, Jeremias Braid

**Affiliations:** Department of Psychology, Centre for Cognitive Neuroscience, Paris-Lodron-University of Salzburg, Salzburg, Austria

**Keywords:** intervention, performance, review, sport, virtual reality

## Abstract

The present article reports a narrative review of intervention (i.e., training) studies using Virtual Reality (VR) in sports contexts. It provides a qualitative overview and narrative summary of such studies to clarify the potential benefits of VR technology for sports performance enhancement, to extract the main characteristics of the existing studies, and to inform and guide future research. Our literature search and review eventually resulted in 12 intervention studies with a pre vs. post design focused on different sports, including target and precision sports (archery, bowling, curling, darts, golf), bat/racquet and ball sports (baseball, table tennis), goal sports (football/soccer, basketball), martial arts (karate), and sport-unspecific processes such as bodily sensations and balancing. The samples investigated in the primary studies included novice, amateur, and expert athletes (total aggregated sample size *N* = 493). Many studies found statistically significant effects in relevant target skills following interventions in VR, often outperforming training effects in passive or active control conditions (e.g., using conventional training protocols). Therefore, interventions in VR (or extended reality) have the potential to elicit real effects in sports performance enhancement through training of motor and psychological skills and capabilities in athletes, including perception-action skills, strategic, tactical and decision-making, responding to unexpected events, and enhancing psychological resilience and mental performance under pressure. The neurocognitive mechanisms (e.g., visual search behavior, imagery), methodological aspects (e.g., adaptive training difficulty), and the issues of real-world transfer and generalizability via which these potential sports-performance-related improvements may occur are discussed. Finally, limitations of the present review, the included studies, the current state of the field in general as well as an outlook and future perspectives for research designs and directions are taken into consideration.

## 1. Introduction

Virtual reality (VR) refers to a simulated experience in a virtual world. It often utilizes VR headsets for pose tracking and 3D near-eye displays to generate the feeling of immersion in the simulated experience. In recent years—driven by technological advancements, increased accessibility, and mobility of VR systems—there has been an enormous interest in VR applications across a broad range of recreational and high-performance domains, including sports (Bailenson, 2018; Greengard, 2019). Specifically, VR can be used for training in motor, cognitive, and mental skills, strategy, and tactics by manipulating environmental constraints (i.e., changing the training conditions) in an immersive virtual environment (Cotterill, 2018; Schack et al., 2020). Along with, e.g., the extent of immersion, this can be regarded as one of the supposed mechanisms at play concerning effectivity of VR training (Gray, 2019; Wood et al., 2021).

Across professional high-pressure, high-performance environments, VR provides interesting new solutions, especially regarding training and learning of complex skills in otherwise costly or dangerous situations. When used properly—i.e., keeping session times short and taking breaks to avoid VR sickness (a.k.a. cybersickness, that is, symptoms resembling motion sickness)—VR allows safe and repeatable training tasks and affords complete control over the training environment including stimuli and difficulty (e.g., Wood et al., 2021). Therefore, it is considered a promising tool for skill acquisition and refinement in sports (Miles et al., 2012; Faure et al., 2019).

Anecdotally, VR technology is already being used by approximately half of the English Premier League (football) clubs, to target specific cognitive skills and simulate matchday pressure (Cunningham, 2022), and to minimize the risk of head injuries during practice (Hart, 2018). Furthermore, VR offers the teams a promising tool during rehabilitation of injuries by providing an immersed feeling of real training and games during injury breaks, which would be absent without VR (Chen et al., 2009; Gokeler et al., 2014; Gumaa and Rehan Youssef, 2019). In particular, during rehabilitation of injured players, the VR environment can enable a well-controlled and relatively safe environment with respect to physical demands (compared to training in the real world), while at the same time providing potential powerful cognitive stimulation and intense mental training. In addition, VR may be used for the evaluation of athlete injuries and movement patterns in a safe and well-controlled virtual environment (e.g., Buoite Stella et al., 2022; Deodato et al., 2023).

In addition, youth and academy players might benefit from VR, as simulations of loud fan chants or in-match pressure situations (e.g., opponents running toward them at a high pace) are designed to prepare them to deal with stressful situations in senior football at an early age. As recently shown, the presence or absence of spectators in a football stadium has substantial effects on the psychological states, behavior, and performance of football players and teams (Leitner and Richlan, 2021a,b; Leitner et al., 2022; Richlan et al., 2023). This may hold even more true for less experienced and younger players, who therefore might benefit even more from this kind of VR training.

Apart from the aforementioned applications, VR technology can also be used to train visual awareness of the players’ surroundings in the form of scanning behavior (e.g., on the football pitch) (Fortes et al., 2021; Be Your Best, 2023; REZZIL|Rezzil - Cognitive Development and Analysis, 2023). Improving one’s scanning behavior might be especially desirable for football players in central playing positions, since—at the professional level—they scan the most and a higher scanning frequency is linked to a higher probability of pass completion (Jordet et al., 2020).

The present review aims at providing a qualitative overview and narrative summary of VR (and extended reality) intervention studies in sports. The extraction of the main characteristics and results of the existing studies provides the foundation for a scientific evaluation of the state-of-the-art in this field and may be used to inform and guide future empirical research. The clarification of potential behavioral performance effects following VR training is important to better understand the specific ways that VR can be used to enhance motor and psychological skills and capabilities in athletes, including perception-action skills, strategic, tactical and decision-making, responding to unexpected events, and to enhance psychological resilience and mental performance under pressure. In addition, limitations of current studies and future perspectives will be discussed. A main goal of the present review article is to inform and to guide future research designs and directions and to facilitate systematic progress in this relatively novel field of research.

VR training enables athletes of various sports to engage in learning, practice, and rehearsal that in real life can be physically demanding, dangerous, impractical, or otherwise expensive (with respect to human, technical, or temporal resources) (Wood et al., 2021). Provided that the virtual sports environment evokes experiences and psychological states that are the same as—or similar to—the real sports environment, the transfer of skills from VR to the real world should be possible (Kim et al., 2019).

Consequently, if training drills in VR are a true and convincing representation of the skills needed in the real world, those that excel at the sport in the real world theoretically should also perform well in the virtual world (Gray, 2019). For example, evidence from an early study with football (a.k.a. soccer) goalkeepers, whose task was to catch curved free kicks, partially supports this notion of a performance-related differentiation between elite and non-elite athletes in VR, with the most experienced goalkeeper at the adult national level showing the best performance in a difficult task in VR (Dessing and Craig, 2010). To successfully design VR applications that represent real-world sports task settings, however, one needs to understand how skills are acquired.

Skill acquisition (or motor learning and control) investigates the performer-environment interplay involving perception, intention, calibration, and action. It can further be described as a voluntary act to solve a certain motor skill problem and achieve a specific goal (Magill and Anderson, 2010). Thus, movements of joints and body segments are controlled voluntarily. In sum, activation and coordination of muscles and limbs by the neuromuscular system—in the context of performance—is a central matter of investigation in the science of skill acquisition.

Motor learning is often described as a process that can be divided into distinct stages (Araújo and Davids, 2011). For example, Fitts and Posner (1967) first proposed a model of motor learning consisting of three stages. The first stage was termed the “cognitive stage,” in which learners focus on the “what” and “how” of the task. This includes maintaining high attention regarding specific movements and receiving feedback from another person (e.g., the coach).

For instance, a novice football player might want to strike the ball in an optimal way to score a goal on a free kick. The player pays close attention to how the ball is struck and how he moves his body—especially his shooting foot. In addition, he receives feedback from a coach that assesses the curve of the shot. Usually, in this stage, learners make a lot of mistakes and are inconsistent, but experience large performance gains.

The second stage, called the “associative stage,” has been reached when the learner starts to improve performance and associates cues to solve the motor problem. The variability of both errors and performance decreases due to the establishment of the fundamentals. Conscious effort, however, is still required. The third and “autonomous stage,” implies that the performance of the skill has reached automaticity. The performer is not required to fully focus on the task anymore and can engage in another task simultaneously. Furthermore, correcting oneself becomes a big part of this stage, since errors are detected more easily. Therefore, adjusting one’s performance without entirely relying on the feedback of others is possible.

In this context, if VR training is sufficiently realistic to support real-world transfer—that is, the translation of potential training effects in the virtual world to performance increases in the real world—VR can be an extremely helpful tool in skill acquisition (see Section 4.3 for an in-depth discussion on the issues of transfer and generalizability based on the findings of this review). Research into the use of VR suggests that with realistic representations of the main elements of a skill, VR can distinguish between elite and novice with elite athletes performing better in VR (Gray, 2019; Harris et al., 2020).

Based on this, a study investigating the validity of the VR software REZZIL^®^ found that professional football players outperformed academy and novice players on football drills in VR (Wood et al., 2021). Therefore and because of the opportunity to specifically train skills that are associated with potential health concerns in the real world (i.e., headers), this VR software may offer serious benefits for safe skill acquisition and refinement in football.

Importantly, the way simulations are presented substantially influences the outcome. For example, in an early study using computer screens instead of head-mounted displays, a more immersive presence (e.g., point of view vs. bird’s eye view) increased motivation and speed while cycling (IJsselsteijn et al., 2004). Similarly, the presence of other people also has an impact on performance. In this regard, Irwin et al. (2012) were able to show the Köhler effect during a video game-based cycling task. Participants either cycled alone or with a partner. Cyclists in the conjunctive group (who have been told that their team score would be based on the rider who quit the task first) showed higher persistence than those cycling alone or in the coactive group (no team partnership).

More research and evidence from applied practice, however, is required to further clarify the advantages of using VR; and whether the virtual environment is evoking experiences and psychological states that are the same as—or similar to—the real world. Furthermore, evidence of the neuroscientific basis of potential VR training benefits (i.e., in terms of neural plasticity) is largely missing (with notable recent exceptions, e.g., Köyağasıoğlu et al., 2022).

Our review is aimed at providing this evidence to better understand the specific ways that VR can be used to enhance motor and psychological skills and capabilities in athletes, including perception-action skills, strategic, tactical and decision-making, responding to unexpected events, and to enhance psychological resilience and mental performance under pressure. Specifically, the present review aims at providing a qualitative overview and narrative summary of VR (and extended reality) intervention studies in sports and to extract the main characteristics of the existing studies. This provides the foundation for a scientific evaluation of the state-of-the-art in this field and may inform and guide future empirical research.

## 2. Materials and methods

To provide a comprehensive overview of studies using VR interventions aiming at training physical and/or psychological sport-related skills, we conducted a review and narrative summary of the results (Bates, 2022; Gunnell et al., 2022). This is a commonly used method in fields such as psychology, economics and management research to present and synthesize the current state of research, and to inform future research in a particular field (Webster and Watson, 2002; Frank and Hatak, 2014; Fisch and Block, 2018). The search and review procedure was as follows:

We used the search platforms Web of Science, Google Scholar, and PubMed. All sources were last searched on July 4th, 2022.

The following keywords were used across all platforms to conduct various searches:

1. Virtual reality AND athletes
2. Virtual reality AND athletic performance
3. Virtual reality AND exercise
4. Virtual reality AND sport
5. Virtual reality AND sport psychology

In addition, we searched the reference lists of all articles that were considered relevant for the review to identify as many relevant studies as possible. Grey literature such as theses, newspaper articles and comments were excluded (Gunnell et al., 2022). Papers that were not relevant for the scope of the present work (e.g., opinion papers on using VR for athletes) were excluded as well (e.g., Düking et al., 2018).

The literature search and preselection procedure resulted in 51 articles, all of which were read and screened for basic information (authors, year, journal, sport, sample, peer-review status etc.). In addition, the following characteristics and relevant factors were extracted and coded from each article and documented in detail: intervention duration and volume, type of VR system, performance factors and aims, number and properties of dependent and independent variables, results and conclusion/discussion, transfer, and generalizability. Data were sought and documented for all outcomes.

Extraction of study characteristics and the decision whether a study met the inclusion criteria was done first independently and then consensually by all authors. We also sorted the articles by the type of study, differentiating between intervention (training, including transfer), diagnostics/assessment and review papers. In some cases, the exact VR technology used was difficult to assess from the information given in the original articles. We focused our review article primarily on studies using head-mounted displays, but also included VR settings with screens. Whenever a study did not use head-mounted displays, this information is explicitly stated.

Inclusion criteria: Twelve of the 51 preselected articles were finally included in this review based on the study type (intervention study with pre-post comparison) and study quality assessment (critical appraisal; Tod et al., 2022). In particular, the inclusion criteria were (i) peer-review status (only peer-reviewed articles were included) and (ii) journal source (conference papers were excluded). We deliberately decided to focus on intervention (i.e., training) studies with a pre vs. post design to provide a comprehensive overview of the VR interventions and their effects on real-life performance that have been explored up until this point. The results are summarized in the form of a qualitative review including a descriptive analysis and narrative synthesis and are presented in the following section.

## 3. Results

To detect potential effects due to, e.g., varying VR technology, it is important to take differences in study characteristics into consideration. For example, VR device “A” might be more comfortable, capable of displaying in a higher resolution and facilitate immersion to a larger degree than VR device “B.” Furthermore, some sports tasks are more suited for VR training than other sports tasks. This may hold even more true for sports domains that rely on certain external conditions (e.g., the feeling of water surrounding the body in water sports).

Table 1 provides an overview of the 12 intervention studies and shows their main characteristics. There was a great variety in terms of VR systems that were used: 2x screens (not further specified), 2x HTC Vive Head Mounted Display, 2x Utopia 360 Head Mounted Display with an LG3 smartphone (LG Electronics), 2x Head Mounted Display (not specified), 1x virtual (projected) dart board, 1x ColorCross VR goggles, 1x Oculus Rift DK2 Head Mounted Display, and 1x Oculus Go Standalone VR HMD (Facebook Technologies).

**Table 1:** Overview and main characteristics of the 12 intervention studies included in the review.

References	Sport(s)	Sample (male/female; level of expertise; age in years)	Duration	Intervention volume	VR technology	Performance factors and aims	Independent variables	Dependent variables	Key findings	Transfer	Generalisability
Bedir and Erhan (2021)	Curling Bowling Archery	14 curling, 13 bowling, 7 archery (17/17; expert; M = 21.7)	4 weeks	24 h of imagery training	Head Mounted Display, not specified	Determining whether shot performance and imagery skills can be enhanced more by using VR based imagery training programs (VRBI) than by using the most common training model, i.e., visual motor behavioral rehearsal and video modeling (VMBR+VM)	-VRBI-VMBR+VM-Control	-Movement Imagery Questionnaire-Revised (MIQ-R)-Shot performance-Semi-structured interviews with three athletes from each experimental group	-Non-significant increase in average weekly shot performance for VMBR+VM and VRBI groups but not for the control group-Shot performance for the VRBI group started improving earlier and was better than in both other groups during weeks 2-4-Significant improvements of imagery skills for both experimental groups with no significant difference between the two at post-test	x	–
Tirp et al. (2015)	Darts	38 (26/12; novice; M = 25.0)	1 week	Three sessions of 50 throws each	Virtual dart board, projected onto a wall	Exploring whether VR darts practice can improve real-world throwing and vice versa	-VR training-Real-world training-No training (control)	-Quiet eye duration (QED)-Throwing accuracy (radial error)	-Both training groups showed a descriptive, non-significant increase in throwing accuracy; the control group showed a slight decrease-QED increased significantly from pre-to post-test with no significant difference between groups-Both throwing accuracy and QED were significantly better for virtual throwing training compared to real-world throwing training	x	–
Harris et al. (2020)	Golf (putting)	18 (11/7; expert; M = 29.2) 40 (19/21; novice; M = 21.6)	Experiment 1: one day Experiment 2: three days	Experiment 1: 40 putts (40 min; assessment immediately after the VR rehearsal) Experiment 2: 40 putts (45 min; post-test two days after VR or real-world training)	HTC Vive Head Mounted Display	Experiment 1: Investigating the effect of a VR putting warm-up on the subsequent real-world performance of expert golfers Experiment 2: Investigating whether VR putting practice can increase real-world performance and vice versa	Experiment 1: -VR putting warm-up Experiment 2: -VR training-Real-world training	-Putting performance (radial error) -Quiet eye duration (QED)	Experiment 1: -No difference in performance between real-world putts at baseline and following VR putts-Significant decrease in performance for the first real-world putt after VR training; no difference in the subsequent putts-No difference in QED between real-world putts at baseline and following VR putts-Significant reduction in QED for the first real-world putt after VR training; no difference in the subsequent putts Experiment 2: -Significant increase in real-world performance after training but no difference between real-world training and VR training-Significant increase of performance in the virtual environment for the VR group but not for the real-world group-No changes in QED	x	–
Gray (2017)	Baseball (batting)	80 (all male; amateur; 17–18)	6 weeks	Two sessions of 45 min per week (=9 h)	Screen of 2.11 m x 1.47 m	Comparing the effect of different kinds of extra VR trainings on results in performance tests as well as the future baseball career to the effect of extra real-world training and no extra training	-Adaptive training in a batting VR-Extra sessions of batting practice within the VR-Extra on-field sessions of real batting practice-No additional practice (control)	-VR batting test, real batting test and pitch recognition test: Eight specific measures were derived from those tests (e.g., number of hits in VR batting test or number of correctly identified pitches) -On-base percentage (OBP) for the high school season following the training	-VR adaptive group had significant pre-post improvements for all eight measures -Real-world batting practice group showed significant pre-post improvements for 7/8 measures -VR batting practice group showed significant improvements for 3/8 measures -Control group showed significant improvements for 2/8 measures -OBP was significantly higher for the VR adaptive group than for both the VR batting practice group and the control group and marginally higher than for the real-world batting practice group -Number of participants that played at least one season at a higher level than high school: VR adaptive = 8, VR batting practice = 1, real-world batting practice = 3, and control = 1	x	- (not explicitly reported, but probable)
Ross-Stewart et al. (2018)	Baseball	27 (22 eligible for analysis) (all male; expert; M = 19.8)	12 weeks	12–15 min per day, one to two times a day, eleven sessions per week on average (=26–33 h)	ColorCross VR goggles	Exploring whether imagery-related VR interventions increase the ability and tendency of D1 baseball players to use imagery-related skills during practice and competition	-Using VR imagery training (frequency of use decided by the players)	-Sporting Imagery Ability Questionnaire (SIAQ) -Test of Performance Strategies (TOPS)	-No difference between those who used the intervention <7 times a week and those who used it more -Significant improvements in skill imagery, goals imagery, mastery imagery, practice automaticity, practice relaxation, practice self-talks, practice imagery, competition automaticity, self-talk competition, imagery competition, and negative thinking competition	x	x
Michalski et al. (2019)	Table tennis	57 (34/23; novice; M = 21.8)	4 weeks	Seven sessions of 30 min (=3.5 h)	HTC Vive Head Mounted Display	Exploring whether VR table tennis practice improves performance in real-world table tennis compared to a group with no practice at all	-VR training-No training (control)	-Qualitative performance evaluation based on serving accuracy and rallying tasks -Quantitative performance evaluation based on ball height, strength, consistency, technique, and coordination	-Significantly larger improvements in both quantitative and qualitative measures for the VR group compared to the control group -While 93.1% of participants in the VR group showed improvements, 85.7% of participants in the control group also showed improvements	x	- (not explicitly reported, but probable)
Fortes et al. (2021)	Football (soccer)	26 (all male; national level; M = 15.4)	8 weeks	18 sessions with 20 video clips (M = 10.2 s) per session (=1 h)	Utopia 360 Head Mounted Display with an LG3 smartphone	Comparing the improvements of young football players with a VR training to the improvements in perceptual-cognitive skills (passing decision making, visual search behavior, inhibitory control) with a video-simulation screen training	-VR training group -Video-simulation screen training group (VID)	-Passing decision-making (appropriate vs. inappropriate decisions) -Visual search behavior -Inhibitory control performance	-Both training groups significantly improved on all three variables with the VR group showing significantly greater improvements than the VID group for passing decision-making and visual search behavior -No difference was observed for improvements of inhibitory control	x	x
Nambi et al. (2020)	Football (soccer)	45 (all male; amateur university players; 18–25)	4 weeks	Five sessions of 30 min per week (=10 h)	Screen, not specified	Comparing the effect of VR training on clinical (pain, wellness) and athletic performance (sprinting, jumping) of university football players with chronic lower back pain to the effects of isokinetic and conventional training	-VR training-Isokinetic training (IKT) -Conventional training (control)	-Pain intensity: visual analog scale (VAS) -Sprint performance: 40 m dash, 4×5 m sprints, submaximal shuttle running (5 min, 20 m, 12 km/h average speed) -Jump performance: countermovement jump, squat jump	-All groups showed considerable improvements in pain intensity and player wellness, however, the VR group had significantly higher improvements than the other two at all follow-up measurements -All groups improved significantly in sprint performance with VR and IKT improving significantly more than control and VR improving slightly more than IKT -All groups improved significantly in jump performance but there were tendencies for larger improvements for the VR group	x	x
Pagé et al. (2019)	Basketball	27 (21/6; amateur; M = 19.4)	1 week	Four sessions of 50 short video clips (M = 14.7 s) between days 2 and 6 (=49 min)	Utopia 360 Head Mounted Display with an LG3 smartphone	Comparing the effects of watching video clips of basketball plays (both trained and untrained ones) in VR on decision-making skills to the effects of watching the videos on a computer screen	-Watching in VR -Watching on computer screen (CS) -Watching NCAA playoff games on computer screen (control; CTRL)	-Virtual decision-making at the end of each video clip -Pre and post on-court decision-making for 18 plays (12 trained, six untrained)	-Significant increase of decision-making accuracy in training over time but no significant difference between groups -For trained plays, VR and CS groups significantly outperformed CTRL in the on-court post-test; there was no significant difference between VR and CS, but a tendency for VR to perform better (79.0% decision-making accuracy vs. 73.2%) -For untrained plays, VR significantly outperformed both CS and CTRL during the on-court post-test (78.9% vs. 60.9 and 60.2%); there was no significant difference between CS and CTRL	x	x
Petri et al. (2019)	Karate kumite	15 (10/5; amateur; 13–17)	32 weeks	Ten sessions of 10–15 min spread across 6 weeks (=100–150 min)	Oculus Rift DK2 Head Mounted Display	Exploring whether sport-specific VR reaction training can improve the response behavior to upcoming attacks in karate athletes	-Normal training + VR training -Normal training only (control)	-Pre-and post-tests: Two sports-unspecific reaction tests of the Vienna Test System (S1 and S4), karate-specific movement analysis (pre-tests were compared with a group of 14 non-karatekas) -Performance analysis: time for response, response quality, and response type	-No significant differences in sports-unspecific reaction time (but there were small improvements) -Significant improvements in time for response for all attacks -Significant improvements in quality of response for all attacks	–	–
McClure and Schofield (2019)	Cycling	29 (N/A; novice; college students older than 18)	1 day	Two sessions of 6 min (=12 min)	Head Mounted Display, not specified	Exploring the effects of wearing a VR HMD on heart rate and other bodily sensations during exercise on a stationary bike compared to not wearing one	-VR while cycling -No VR while cycling	-Heart rate-Body sensations questionnaire (respiratory, cardiac, arousal/anxiety, gastro-intestinal, dizziness, and pain) -Satisfaction questionnaire	-Participants’ heart rate during exercise was significantly higher when wearing the VR HMD -Perceived bodily sensations were significantly higher in the VR condition, however, most participants (62%) reported feeling like they could continue exercising longer when wearing the HMD -Over 50% of participants stated that they preferred the VR condition and were able to focus better	–	–
Köyağasıoğlu et al. (2022)	Balance skills	57 (29/28; novice; M = 21.7)	4 weeks	Three days per week, 30 min each (=6 h in total)	Oculus Go Standalone VR HMD, Facebook Technologies	Comparing the effects of mental training in different visual environments on balance skills and simultaneously investigating the prefrontal cortex responses using fNIRS	-VR mental training -Conventional mental training -No training (control)	-Stabilometry tests -Star Excursion Balance Test (SEBT) -Prefrontal hemodynamic activity (fNIRS)	-Stabilometry: significant improvement for at least one variable in the VRMT and CMT groups only -SEBT: (a) Composite reach distance: significantly increased in both mental training groups, significantly decreased in the control group, (b) Separate directional scores: reach distance significantly increased in both mental training groups for nondominant leg posterolateral and posteromedial, and dominant leg posterolateral; significant increase in nondominant posteromedial only for VRMT, and (c) Between-group comparisons: improvements in dominant leg posteromedial and posterolateral significantly higher than in the control group for VRMT and CMT groups; nondominant leg improvements significantly higher than control group for VRMT only -fNIRS: no significant change in oxyhemoglobin levels during stabilometry assessment; oxyhemoglobin levels significantly reduced during SEBT for the control group only	x	–

Most studies (9 out of 12) employed head-mounted displays. Although qualifying as VR, the three studies using projectors or conventional screens (Tirp et al., 2015; Gray, 2017; Nambi et al., 2020) should be considered separately from the studies using headsets. One might speculate, that these studies fall more into the spectrum of “extended reality” (Bailenson, 2018; Greengard, 2019). Therefore, the results and implications of these studies will be treated with high caution.

Intervention duration varied greatly across studies and ranged from 1 day to 32 weeks (*median* = 4 weeks). One third of the studies, however, used an intervention duration of 4 weeks. Concerning total intervention volume, we observed a minimum of 12 min and a maximum of 29.50 h (*median* = 2.79 h) across the studies included in this review. Note that we excluded the study of Tirp et al. (2015) in the volume calculation since no exact total time could be calculated.

In sum, this review involves data of *N* = 493 participants (total aggregated sample size). Studies’ sample sizes ranged from *N* = 15 (Petri et al., 2019) to *N* = 80 (Gray, 2017). The included samples can further be described by the participants’ level of expertise. Four studies included novices only, five investigated amateurs only (including national-level youth players), and two involved experts only. Furthermore, one study included both novices and experts (Harris et al., 2020). Seven studies included both males and females, four studies included only males, and one study did not provide information concerning sex of the participants.

Regarding the investigated sports domains, a large variance could be observed. Across all studies, two types of sport were investigated twice: baseball and football (soccer). The sports domains involved curling, bowling, archery, darts, golf, baseball, table tennis, football (soccer), basketball, karate, cycling, and balance skills. The studies were categorized based on their sport-specific elements and their results are described in detail in the following subsections.

### 3.1. Target and precision sports (e.g., archery, bowling, curling, darts, golf)

Bedir and Erhan (2021) examined the effects of a VR-based imagery training program on shot performance and imagery skills in target-based sports and compared it to a visual motor behavior rehearsal and video modeling training program as well as to a control group. Thirty four elite athletes playing target-based sports (14 curling, 13 bowling, 7 archery) took part in this four-week intervention study. They were randomly assigned to one of three groups: VR-based imagery training (VRBI), visual motor behavioral rehearsal and video modeling (VMBR+VM), control group.

Performance evaluations were prepared by experts and each participants’ score was obtained by using the weekly average of their daily performance scores. Additional data was collected by using the Movement Imagery Questionnaire-Revised (MIQ-R) (Hall and Martin, 1997), a scale to determine the imagery ability of athletes, as well as semi-structured interviews with three participants each from the intervention groups.

Results showed larger improvements by the VRBI training group for both shot performance and imagery skills, compared to the visual motor training program and the control group. Athletes in the VRBI training group were able to adapt to the new imagery training faster than the other intervention group which could be observed via the larger increase in shot performance. These findings point toward the potential of VR-based imagery training programs being more effective than the most widely used current methods.

In a seminal one-week intervention study using a wall-projection of a dart board (no head-mounted display), Tirp et al. (2015) compared the effects of VR training to real-world training on dart-throwing accuracy. Thirty eight participants who were all inexperienced in throwing darts were randomly assigned to either VR training, real-world training, or no training at all. Training consisted of three sessions with 50 throws each. In addition to throwing accuracy, which was assessed for both virtual dart-throwing and real dart-throwing, quiet eye duration (QED) was assessed both before and after the intervention.

Results revealed a descriptive, non-significant increase in throwing accuracy for both training groups and a slight decrease for the control group. For QED, there was a significant increase for all groups with no significant differences between the groups. Both throwing accuracy and QED were significantly better for virtual throwing compared to real-world throwing. Overall, although there were some limitations concerning the interpretation of the results (e.g., missing data on dart throwing kinematics), this study points toward successful transfer from VR (or extended reality) to the real world not only for motor performance but also for the underlying mechanistic perceptual processes.

Focusing on golf, the short-term (i.e., 1 day) intervention study by Harris et al. (2020) included two separate experiments: experiment one examined the effect of 40 VR warm-up putts on real-world putting performance and quiet eye duration (QED) in 18 expert golfers. Experiment two examined the effects of either VR or real-world putting training on real-world and virtual putting performance and QED for 40 novice golfers who were randomly assigned to one of the two training groups.

Results of experiment one (experts) showed no significant differences between putts at baseline and putts after VR practice. There was, however, a significant decrease in performance as well as in QED only for the first real-world putt after VR training. Results of experiment two (novices) showed a significant increase in real-world performance after training, but no difference between VR and real-world training.

There was also a significant increase in virtual putting performance after training, but only for the VR group. No main effects or interactions were found for QED. One might speculate, that the findings of experiment one indicate the potential of VR warm-ups disrupting already finely tuned visuo-motor skills in expert golfers and therefore point toward potentially detrimental effects of VR practice. In contrast, the findings of experiment two point toward the potential of VR training of visuo-motor skills being of similar efficiency as real-world training in novice golfers.

### 3.2. Bat/racquet and ball sports (e.g., baseball, table tennis)

One of the first intervention studies (Gray, 2017) explored the effects of extended reality baseball batting practice on performance measures in the virtual environment (using a projection on a conventional screen, no head-mounted display) as well as in the real-world. Eighty high-school baseball players were randomly assigned to either of four conditions/groups: adaptive hitting training in VR, extra sessions of batting practice in VR, extra sessions of real batting practice or no additional practice. In adaptive hitting training, speed, crossing height, and lateral location of the pitch were matched to the batter’s skill level by slightly varying each parameter at the beginning of each session and adjusting it based on batting performance.

Each type of training consisted of two 45-min sessions per week over the course of 6 weeks. Performance was assessed with a VR batting test, a real-world batting test and a pitch recognition test, all three of which were conducted at baseline and after the intervention. Eight specific dependent variables were derived from those tests (e.g., number of hits, number of correctly identified pitches). Performance in competition was assessed by calculating players’ on-base percentage for the season following the intervention as well as determining the highest level of competition at which each player competed for at least one full season.

Results showed statistically significant pre-vs. post-intervention improvements in all eight test measures for the VR adaptive group, in seven out of eight measures for the real-world batting practice group, in three out of eight measures for the VR batting practice group and in two out of eight measures for the control group. In-competition on-base percentage was significantly higher for adaptive VR training than for the three other groups (VR batting practice, real batting practice, and control). In addition, the number of participants who played at a level higher than high school in the season following the intervention was: eight in VR adaptive group, one in VR batting practice group, three in real batting practice group and one in control group.

These results showed the efficacy of using virtual environments by means of extended reality—in particular, individually adaptive VR training—for batting practice by providing evidence for both near and far transfer to the real world. It also seems beneficial to focus on the mechanisms underlying a technique for VR training by individually adapting difficulty (e.g., speed, crossing height, lateral location of pitches) instead of just performing more repetitions in the virtual (or extended) environment.

Ross-Stewart et al. (2018) explored the effects of a three-month VR-assisted imagery skills training program in 27 NCAA Division 1 college baseball players. The program consisted of 360-degree videos of situations that athletes would be in before and during competition along with scripts that helped the athletes in imagining themselves being successful and in developing routines. Study participation was voluntary and out of the 27 only five did not take part in the full program, leaving 22 participants for data analysis. The use of psychological skills and strategies was assessed before and after the intervention period.

Results showed significant improvements in skill, goals, and mastery imagery as well as in the ability and use of several other psychological strategies (e.g., automaticity, self-talk, imagery) during practice and competition. These results show that VR-assisted imagery training can be an effective method of improving sports-related psychological skills, including both cognitive performance and mental strategies. It should also be noted that the program received very positive feedback from both players and coaches, as reflected in the high compliance rate.

Michalski et al. (2019) explored the transfer of VR table tennis practice to the real world. Fifty seven novice participants were randomly assigned to either the VR training group or the control group who received no training. Real-world performance was assessed by an expert (international medals, 40 years coaching experience) before and after seven 30-min VR sessions spread across 4 weeks (total training volume = 3.5 h).

Results showed significantly larger improvements in both quantitative (e.g., serving accuracy and rallying tasks) and qualitative (e.g., ball height, strength, consistency, technique, and coordination) measures of performance for the VR group compared to the control group. In sum, 93% of participants in the VR group and 86% of participants in the control group showed improvements. Based on these findings it seems that skills learned in VR can indeed be transferred to the real world. It should be noted that this study focused on novice table tennis players and that it is an open question whether VR training might also be useful for highly skilled competitive players.

### 3.3. Goal sports (e.g., football/soccer, basketball)

Fortes et al. (2021) examined the effects of watching short first-person videos of two distinct offensive patterns in football (soccer) either in VR or on a video screen. The participants were 15-year-old football players competing at the national level (*N* = 26; mean experience in playing football = 5.0 years) who were randomly assigned to one of the two conditions prior to the eight-week intervention.

The dependent variables were (a) passing decision-making performance, which was assessed using standardized coding criteria during small-sided games (either 3 vs. 3 or 5 vs. 5 players), (b) visual search behavior, which was assessed using portable eye-trackers during those small-sided games, and (c) inhibitory control, which was assessed using a computer-based Stroop task.

Results showed greater improvements in passing decision-making performance and visual search behavior for the VR group compared to the video screen group. There was no group effect for inhibitory control, as both groups showed similar increases in performance. These findings point toward the potential benefit of VR-assisted training of perceptual-cognitive skills in young football players being more effective than more traditional video screen-assisted training.

Nambi et al. (2020) compared the effects of 4 weeks of VR training (using a conventional screen, no head-mounted display) on clinical outcome and sports performance of 45 university football (soccer) players with chronic lower back pain to the effects of isokinetic training and conventional training. All measurements (pain intensity, player wellness, sprint performance, and jump performance) were conducted at baseline as well as 4 weeks, 8 weeks, and 6 months after intervention. Results showed significant improvements in pain intensity and player wellness in all three groups, but VR training showed considerably higher improvements than both other groups at all follow-up measurements.

In addition, extended reality and isokinetic training showed significantly larger improvements than conventional training for sprint performance. All groups showed significant improvements for jump performance with VR and isokinetic training showing significantly higher improvements than conventional training. VR training showed tendencies for larger improvements than isokinetic training for both sprint and jump performance. The authors suggested that the slight advantages of extended reality training might be due to increased activity of the human sensory system as well as the activation of experience-related brain plasticity through the changing environment in VR—although immersion was limited due to the use of a conventional screen instead of a head-mounted display.

In a remarkable short-term (i.e., one-week) intervention study, Pagé et al. (2019) explored the transferability and generalizability of improvements after VR-assisted training. Twenty seven basketball players were randomly assigned to either watch pre-determined variations of two distinct offensive patterns in VR or on a computer screen (CS) or to watch NCAA playoff games (top-tier college basketball) on a computer screen (control) for a total of four training sessions during the intervention week.

Participants’ virtual performance was assessed (only for VR and CS) by asking them to decide at the end of each observed play about where they should move next. Participants’ on-court performance was evaluated before and after the intervention by running trained as well as untrained plays, having them decide in the end, and calculating the decision-making accuracy score post-hoc.

Results showed a significant increase in virtual performance over time for both VR and CS with no statistically significant difference between the two. In on-court decision-making for trained plays, VR and CS significantly outperformed the control condition. In contrast, for untrained plays, VR resulted in significantly better decision-making accuracy than CS and the control condition. These results demonstrate that while skills learned in both VR and CS can be transferred onto the court, VR seemed to lead to more generalizable gains regarding decision-making.

### 3.4. Martial arts (e.g., karate)

In this long-term intervention study (32 weeks), Petri et al. (2019) examined the effects of training against a virtual opponent on sport-specific and nonspecific response behavior of 15 experienced young karate athletes. The participants were randomly divided into groups A and B. During the first phase, group A did the VR training (in addition to conventional training) and group B acted as the control group (conventional training only). After a four-month wash-out period the same intervention was conducted again in the second phase with group B as the experimental (conventional and VR training) and group A as the control group. Each phase lasted 8 weeks and consisted of 10 VR training sessions with performance being tested in weeks 1 and 8.

Results revealed no significant effects for sport-nonspecific response behavior. In phase 1, group A showed significant improvements with large and medium effect sizes in time for response and response quality while group B only improved in time for response and with a smaller effect size. In phase 2, group B showed significant improvements with large effect sizes in time for response and response quality while no significant differences were observed for group A. Interestingly, group A managed to maintain their improved performance over the 4-month wash-out period. These results show that VR training can lead to long-term improvements in the response behavior of young karate athletes.

### 3.5. Bodily sensations and balancing

McClure and Schofield (2019) compared the effects of riding an exercise bike at medium effort level on heart rate and perceived bodily sensations while wearing a VR headset to completing the same workout without a VR headset. Twenty nine college students participated in this within-subject design study. During the VR condition, the participants were exposed to outdoor scenery via visual stimulation (e.g., riding down a sidewalk).

Results showed significantly higher heart rate as well as perceived bodily sensations (respiratory, cardiac, arousal/anxiety, gastro-intestinal, dizziness, pain) during the VR workout. Most participants also reported preferring VR and feeling like they could focus better and exercise longer in the VR condition despite their higher heart rate. Although the findings contradicted the authors’ original hypothesis that VR would enhance workouts by distracting participants from their bodily sensations, VR still seemed to lead to longer workouts due to more focus and satisfaction as well as more burned calories due to the higher heart rate.

Conducting a randomized controlled trial, Köyağasıoğlu et al. (2022) investigated the effects of VR training on balance skills and prefrontal hemodynamic responses (assessed via functional near-infrared spectroscopy; fNIRS). Twenty eight females and 29 males were randomly assigned to one of three groups: virtual reality mental training (VRMT), conventional mental training (CMT), and a control group. Age ranged from 18 to 25 years. The intervention lasted for a total duration of 4 weeks (training volume = 3 days per week, 30 min each).

While the VRMT group trained using VR head-mounted displays, the CMT group used conventional computer screens to practice balance with videos containing action observation and motor imagery. Balance skills were assessed at baseline and the end of the intervention via stabilometry and the Star Excursion Balance Test (SEBT). Simultaneously, fNIRS was applied.

Concerning the stabilometry assessment, significant improvements for at least one variable could be observed for the VRMT and CMT groups only. While composite reach distance (i.e., an average measure for all three reach distances) significantly increased in both experimental groups, a significant decrease was observed in the control group for the SEBT. Additionally, for separate directional scores in the SEBT, reach distance significantly increased in the VRMT and CMT group for nondominant leg posterolateral and posteromedial, and dominant leg posterolateral. Nondominant posteromedial only increased significantly for the VRMT group.

SEBT between-group comparisons revealed that improvements in dominant leg posteromedial and posterolateral were significantly higher than in the control group for both mental training groups. For the VRMT group only, nondominant leg improvements were significantly higher than in the control group. While no significant change in oxyhemoglobin levels could be observed during the stabilometry tests, a significant reduction occurred during SEBT (control group only).

## 4. Discussion

The present review revealed that interventions in VR have the potential to elicit real effects in sports performance enhancement. Considering the findings of the included studies, VR training groups consistently outperformed control groups and—in some studies—even showed superiority compared with conventional active training protocols (e.g., computer screens). In line with the idea of a “realist review” (Hunter et al., 2022), some of the neurocognitive/psychological mechanisms and methodological aspects and conditions via which these sports-performance-related improvements may occur are discussed in the following subsections. It should be noted that many of the conclusions remain speculative due to the limited number of available VR intervention studies in the sports context.

### 4.1. Neurocognitive mechanisms: visual search behavior

Visual search behavior (VSB) is an important aspect of athletic performance. The characteristics of what is ideal visual search behavior vary greatly between different kinds of skills. For targeting skills like free throw shooting, putting, throwing darts, or shooting penalties, a longer quiet eye duration (QED), referring to the last fixation on the target before beginning an action, is generally associated with better performance (Vickers, 1996; Vine et al., 2011).

In contrast, increased performance during dynamic situations, such as in a football (soccer) game is facilitated by many fixations of short duration to important locations (i.e., teammates, open space)—so-called scanning behavior—on the pitch (Jordet et al., 2020; Roca et al., 2021). What characteristics of VSB are ideal in certain situations and sports contexts is usually determined by comparing experts to novices and amateurs.

Improving sports-specific VSB is highly desirable. Previous research has shown that different athletic skills can be improved by deliberately training VSB, including saving and shooting penalty kicks in football (Savelsbergh et al., 2010; Wood and Wilson, 2011), anticipating setting direction in volleyball (Piras et al., 2014), putting (Vine et al., 2011), and free throw shooting (Harle and Vickers, 2001). Additionally, Adolphe et al. (1997) observed that elite volleyball receivers who had received VSB training still showed greater accuracy than other receivers that were among the best in the world 3 years after the intervention, indicating long-term effects of such training.

VR automatically and implicitly (i.e., without deliberate practice or explicit instructions) improves complex VSB. VR can help improve the training of VSB in several ways. Fortes et al. (2021) showed that watching short clips in VR led to significant improvements in VSB as well as passing decision-making compared to watching the same clips on a normal screen. Participants showed more fixations of shorter duration toward more suitable locations which is associated with the ability to find more creative solutions and the ability to detect teammates in threatening positions earlier in the play (Roca et al., 2018). These improvements were observed even though the participants had not received any instructions about their VSB.

Similar results were found by Pagé et al. (2019), who observed that while participants who watched short clips of offensive basketball plays either in VR or on a screen improved similarly in decision-making for trained plays, only the VR group showed significant improvements for untrained plays. Due to the similar nature of the two studies as well as the underlying similarities of football and basketball, however, it seems reasonable to hypothesize that the superior decision-making during untrained plays can be partially attributed to VR-induced changes in VSB, similar to those in Fortes et al. (2021).

Under pressure, VSB often changes in a way that is associated with decreases in performance (Behan and Wilson, 2008; Wilson et al., 2009; Vine and Wilson, 2011; Wood and Wilson, 2011). These impairments are consistent with attentional control theory (Eysenck et al., 2007), which states that anxiety both decreases attentional control and increases attention to threat-related stimuli. As mentioned in the Introduction, especially youth and unexperienced players might benefit from VR training, as simulations of in-match pressure situations can help to prepare them to deal with stressful situations in real life senior (professional) football.

Furthermore, Stinson and Bowman (2014) found that VR can induce increased anxiety (both physiological and subjective measures) in a virtual environment where participants had to save football penalty kicks. There is, however, still a need for studies investigating whether VR resiliency training in anxiety-inducing virtual environments can lead to a long-term reduction in sport-related anxiety.

Nevertheless, we speculate that VR training of VSB with increased sport-induced anxiety could have even greater benefits for performance in competitive high-pressure settings, considering that normal training and familiarization has already been found to result in better VSB during performance under pressure and, in turn, better overall performance (Vine and Wilson, 2011).

Different levels of expertise are associated with different characteristics in VSB (for a review see Vine et al., 2011). Previous research has already shown that training of VSB can increase performance even in athletes with already high levels of expertise (e.g., Adolphe et al., 1997). In addition, a study by Vine et al. (2011) found that a single brief quiet-eye training can significantly increase the putting performance as well as QED of elite golfers. Therefore, the efficacy of using VR in the context of training VSB in elite athletes is worth exploring further.

According to Vine et al. (2011), quiet-eye training improves goal-directed attentional control (Corbetta and Shulman, 2002; Corbetta et al., 2008) and strengthens the neural systems for integrating visual information and motor commands to enable efficient motor planning and preparation (Janelle et al., 2000; Land, 2009). The analysis of the neurocognitive mechanisms underlying potential VR training benefits by means of functional and/or structural neuroimaging would be a major step forward in this field of research. The fronto-parietal attentional system in the brain is a promising prime candidate for such intervention-related neuroplasticity.

### 4.2. Neurocognitive mechanisms: imagery

In one of the articles included in this review, Bedir and Erhan (2021) found that VR-assisted imagery training for target sport athletes led to similar increases in performance and imagery ability as the most common training model, that is, visual motor behavior rehearsal and video modeling. Similar results were obtained by Köyağasıoğlu et al. (2022), who compared the effects of VR-assisted imagery training on performance in balance exercises to those of conventional imagery training. Ross-Stewart et al. (2018) observed significant improvements in many different subscales of imagery but did not compare their VR-assisted imagery intervention to a conventional one.

All three studies used the PETTLEP approach to motor imagery by Holmes and Collins (2001). PETTLEP stands for Physical, Environment, Task, Timing, Learning, Emotion, Perspective, which is the checklist of guidelines for designing an imagery intervention. In contrast to traditional approaches to imagery in sport psychology, PETTLEP proposes that physical practice and imagery are located on a continuum and that imagery interventions will be more successful, the closer they are to physical practice. A wide array of studies have shown the efficacy of using the PETTLEP approach to improve performance in sports (Wright and Smith, 2007, 2009; Afrouzeh et al., 2013).

If it is beneficial to design imagery interventions that closely resemble physical practice, the potential of VR to closely simulate reality could be very useful. This could improve the environment aspect of PETTLEP which would then also improve other aspects like emotion, since it has already been shown that VR can induce competition-like emotions such as anxiety (Stinson and Bowman, 2014).

VR has a huge potential for supporting imagery. Due to its 360-degree nature and responsiveness to head movements, VR is more immersive than videos watched on screens, which is currently the most common way to assist athletes in imagery interventions. Assuming an enhanced sense of presence is beneficial to imagery, athletes should be able to image more effectively using VR. VR can also assist athletes in using the ideal imagery parameters. Holmes and Calmels (2008) discuss several of the parameters of imagery, including first person perspective versus third person perspective and the different viewing angles that can be employed in third person perspective. A short video clip played in VR at the start of imagery sessions could let athletes see how they should image, letting them continue the protocol on their own afterwards.

Furthermore, motor imagery in VR allows the use of feedforward modeling with varying degrees of expertise. In a recent study, Frank et al. (2022) performed 3D scans of participants and created avatars that were consequently shown in a virtual environment. These avatars were observed by the participants in either performing a squat that has been executed before or a skilled (simulated) squat. Simultaneously, sensations and feelings linked to movement execution should be imagined.

Results showed advantages for the skilled condition (e.g., in error prevention). The authors conclude that combined imagery and observation of prospective action states support the implementation of cognitive requirements for enhanced motor performance. Therefore, VR can be regarded as a promising tool for the use of learning environments superior to participants’ initial skill level.

### 4.3. Methodological aspects: transfer and generalizability

“Transfer” refers to the question of whether potential training effects in VR can be translated to performance increases in the real world and “generalizability” refers to the question of whether potential training effects in VR can be translated to situations that were not trained in VR. Ideally, one would have VR training protocols that result in or facilitate both, high transfer and high generalizability.

These two aspects (transfer and generalizability) are especially relevant from a practical application perspective. As pointed out previously (e.g., Michalski et al., 2019), they are the two key components of evaluating the effectiveness of VR interventions. In the past, outside of the sports domain, effectiveness has been shown for surgeons who received simulator training and improved in real-world task performance (e.g., Haque and Srinivasan, 2006; Frederiksen et al., 2019).

Although 10 out of the 12 VR intervention studies summarized in this review (and characterized in Table 1) assessed real-world transfer, there is still a lack of systematic evidence regarding the factors underlying transfer of skills trained in virtual environments to real-life sports. Therefore, researchers should aim at identifying the specific relevant levers responsible for increasing the probability of successful real-world transfer.

Transfer of potential training effects from the virtual to the real world can be achieved by considering two different important factors: (i) validity (i.e., the extent to which features of the virtual environment resemble real-world features) and (ii) fidelity (i.e., the similarity between states and behavior elicited by the real world and the VR application) (Wood et al., 2021).

According to Gray (2019), fidelity can be further subdivided into (a) the similarity of emotional experiences (affective fidelity), (b) the similarity regarding task appearance (physical fidelity), (c) the similarity of required perceptual-cognitive skills (psychological fidelity), and (d) the similarity of movements needed to solve the tasks (biomechanical fidelity). Ensuring a high fit between virtual and real-world conditions—through rigorous analysis by researchers and practitioners—is likely to increase the probability of successful transfer (Gray, 2019; Wood et al., 2021).

It must be noted that perceptual features such as perspective (e.g., first person vs. third person) and visualization (e.g., 3D vs. 2D) can also affect real-world transfer (see Casale, 2023). Additionally, achieving the highest possible fidelity might not be the only goal worth striving for. Miles et al. (2012) noted that immersion (or “presence,” i.e., the sense of being there) needs to be evaluated in the context of VR interventions. The prerequisite for systematically increasing presence, however, would be to agree upon a clear definition and a standard method of measurement of this construct.

The second key component in the practical usefulness of VR training is the issue of generalizability. Here, the question is to which extent motor, cognitive, and mental skills, strategy, and tactics trained in the real world can be generalized to untrained situations that the athletes are faced with. This may pertain to situations both inside and outside of VR. Depending on the type of sport, situations can be more or less standardized (i.e., having fewer or more degrees of freedom).

Since only a finite number of situations can be trained, it would be highly desirable to improve the generalizability of VR intervention effects, particularly in the case of team ball sports requiring high flexibility within complex tactical situations (e.g., Faure et al., 2019). In contrast to the issue of real-world transfer, only four out of the 12 VR intervention studies summarized in this review (and characterized in Table 1) took the issue of generalizability into account.

From the participants’ (or trainees’) point of view, a high degree of engagement and motivation, as well as an enriched sensory experience leading to the sense of presence and embodiment might play a decisive role in both aspects of VR training efficacy, that is real-world transfer and generalizability (Tieri et al., 2018). Ultimately, the key is to generate a state of “experiencing.”

Taken together, researchers and practitioners alike should seek a high probability of transfer and generalizability by considering validity, fidelity, perspective, visualization, immersion, engagement, motivation, commitment, and sensory experience. Overall, a high fit between real-world and virtual conditions (Miles et al., 2012) and a high degree of intraindividual variables such as motivation might be the first important step toward doing so.

Nevertheless, assuming that considering the aforementioned factors is sufficient for successful transfer and generalizability might be a misconception. Even though findings from other performance domains such as surgical training suggest real-world performance benefits from VR interventions, it is not guaranteed that this holds true for the sports domain (Harris et al., 2020).

This might be due to a lack of realism regarding movement schemas (i.e., force, timing, body position), and haptic properties (weight of objects, environmental conditions, sensory afferent information) in sports simulators compared to surgical simulators (Miles et al., 2012). Additionally, Harris et al. (2020) suggest that each training outcome should be tested empirically, because the necessary skills differ both between and within sports.

### 4.4. Methodological aspects: limitations and open issues

One limitation of this review in particular and the field in general is the low number of studies and the lack of replication of the studies that were included, which can be explained by the novelty of VR as a technology in the sports context. This means that while many of the current results are very promising, much more research and systematic convergence across studies from independent laboratories and groups is needed to establish and optimize the role of VR within the training of both athletic performance and psychological skills in sports.

Another reason for conducting replications, preferably via pre-registered randomized controlled trials, is to avoid the risk of overestimating the VR training effects by reporting (and publishing) only positive results in favor of the desired effects (publication bias a.k.a. file-drawer problem). As in other disciplines (e.g., medicine), this could overestimate the effectiveness of interventions, which leads to inaccurate scientific evaluation and adverse practical consequences.

Furthermore, future research should be conducted to determine whether different VR (and extended reality) systems and intervention protocols lead to different transfer and generalization effects in real life. This could have great practical benefits through the identification of minimum effective doses and the development and optimization of effective intervention systems and training programs using VR.

Although the heterogeneity of the investigated types of sport demonstrates the broad applicability of training in VR, it complicates the comparison across studies. Similarly, the large range in intervention duration and protocols in the present set of studies (1 day to 32 weeks) makes inter-study comparisons difficult. These first few studies should be considered as a serious starting point, but the field is far from having established standards or even loose guidelines for VR training programs.

A final limitation in the field is the lack of mechanistic evidence on training-induced neuroplasticity across studies. Little is known about the underlying neurobiological mechanisms via which potential sports-performance-related improvements may occur. Future functional and structural neuroimaging studies may help to shed light on this issue. While the VR head-mounted displays make it very difficult to perform neuroimaging during the interventions, it should still be used to explore the neural correlates of any effects of the interventions by means of pre-post measurements (Köyağasıoğlu et al., 2022).

### 4.5. Outlook and future perspectives

At the current state of development and research on VR, it can be seen as a no more, no less promising sports training tool for the future. The findings presented and summarized in this review revealed the potential of VR for sports performance enhancement in the real world. The question remains, whether VR will be widely used by individual athletes, coaches, and sports organizations in the future. To date, VR systems (and their accessories) are becoming more and more powerful, yet at the same time more and more affordable. This will likely accelerate the process of distribution and practical application.

Despite the (up to now) largely weak scientific evidence for its effectiveness, the high modifiability and potential for the creative development of unique solutions count as pro-arguments for investing in VR systems. For example, Frank et al. (2022) applied a creative method by letting participants perceive themselves in the virtual world as performing better than they are in the real world. In the future, original and innovative ideas can be expected more frequently. This is due to the high freedom for creators coming with VR and since research conducted on this topic is only at the beginning.

As shown by Gray (2017), the effects and improvements in sports-related performance can be additionally enhanced if VR training has an adaptive design, that is, the difficulty is adapted to the individual level of expertise. Consequently, the learning effects produced by means of intervention in VR can be greater, which is of high relevance for future research and particularly in high-performance practical applications (e.g., for individual programming of VR training).

As mentioned before, subjective feelings of immersion and presence may play a crucial role in the effectiveness of VR applications, not only in the sports domain. Future studies should assess participants’ perceived immersion and psychological factors such as engagement and motivation during VR sessions to identify potential explanatory variables for the observed effects. It could be hypothesized that participants who perceive less immersion, presence and engagement or motivation during these sessions benefit less with respect to real-life performance gains than those having a richer experience.

Additional recommendations for future studies include the inclusion of well-matched control groups or conditions receiving passive or active treatments. Optimally, this would result in randomized controlled trials (Hariton and Locascio, 2018) with double-blinded group assignment. Ideally such studies would include a variety of both short-and long-term outcome measures to gain a systematic and wholistic picture of potential VR training effects. Such measures should include (i) psychometric tests, (ii) expert (e.g., coaches) ratings, (iii) self-ratings, (iv) subjective experience and motivation, (v) neurobiological (e.g., neuroimaging) measures, and finally (vi) real-life performance measures within the respective sport domain.

## 5. Conclusion

Our review and narrative summary of 12 intervention studies using VR in sports contexts showed many potential applications of this technology for sports performance enhancement. The majority of published studies reported statistically significant training effects following interventions in VR compared with passive or active control conditions (e.g., using conventional training protocols). Due to the heterogeneity of VR (and extended reality) technology used, training protocols, targeted sports, skills, and expertise levels of participants, it is difficult to synthesize, let alone quantify, systematic patterns across studies.

Hence, taking into account the various limitations inherent in the literature, general recommendations for or against the application of interventions in VR in applied sports practice are premature. Nevertheless, some of the assumed underlying neurocognitive mechanisms of potential benefits (e.g., visual search behavior, imagery) are reasonably well understood, making VR an interesting field of research for future neurocognitive studies and a promising technology for training of motor and psychological skills and capabilities in athletes. These potential areas of application include perception-action skills, strategic, tactical and decision-making, responding to unexpected events, and enhancing psychological resilience and mental performance under pressure. Specific recommendations for future research designs and directions are discussed.

## Author contributions

All authors contributed to manuscript conception and preparation, read, and approved the submitted version.
